# Supplementary material for: Retention strategies among those on community supervision in the South: Lessons learned during the COVID-19 pandemic
Source: PLoS One. 2023 Apr 5;18(4):e0283621. doi: 10.1371/journal.pone.0283621 (PMC10075476; doi:10.1371/journal.pone.0283621)

# **S1 Appendix: Study Mailers**

| **Site** | **Holiday/Event** | **What was included with the mailing** | **Frequency** |
| --- | --- | --- | --- |
| All | Valentine’s Day | Sites added candy with the card and study-branded magnetic memo board. | Yearly |
| All | Birthday Card | Card, small swag items (keychain, magnet, etc) | Yearly on Participant's birthday |
| All | Spring | Card, small swag items (keychain, magnet, etc) | Yearly |
| All | Summer | Card, small swag items (keychain, magnet, etc) | Yearly |
| All | Fall | Card, small swag items (keychain, magnet, etc) | Yearly |
| All | Winter/Holiday | Card, small swag items (keychain, magnet, etc) | Yearly |

*Cards are variable, but are bright, attractive, eye catching and seasonally appropriate*

**Selected Examples:**

*Spring Mailer:*


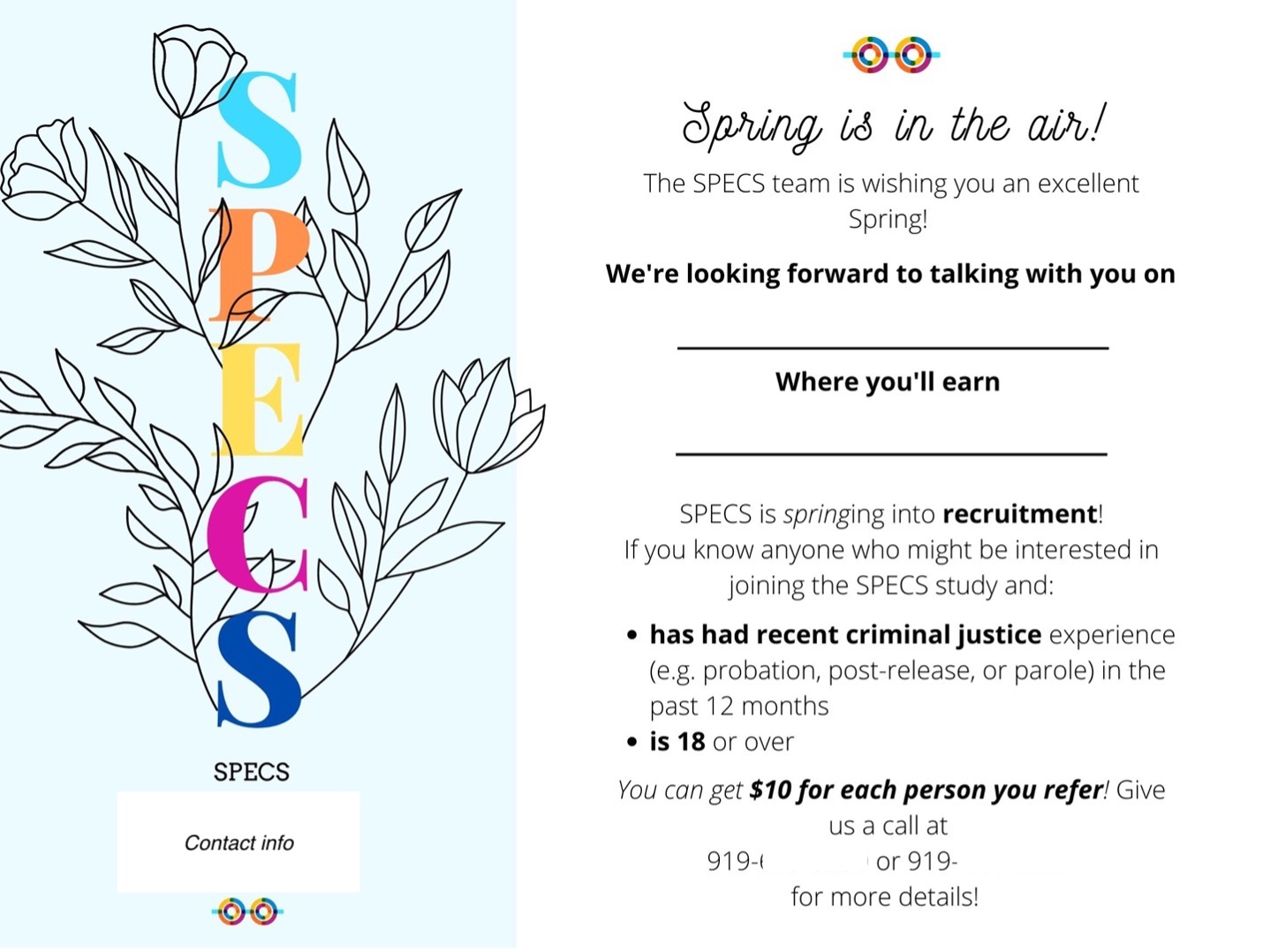


*Summer Mailer:*


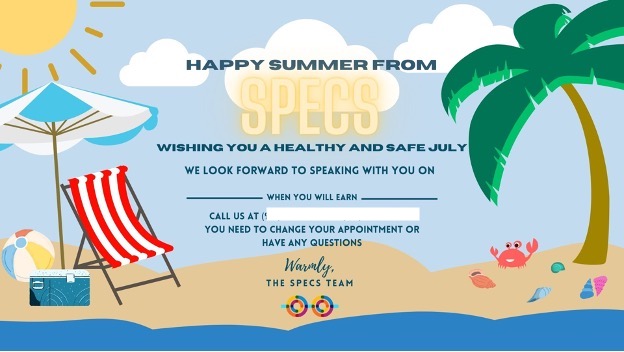


*Valentine’s Day Mailer:*


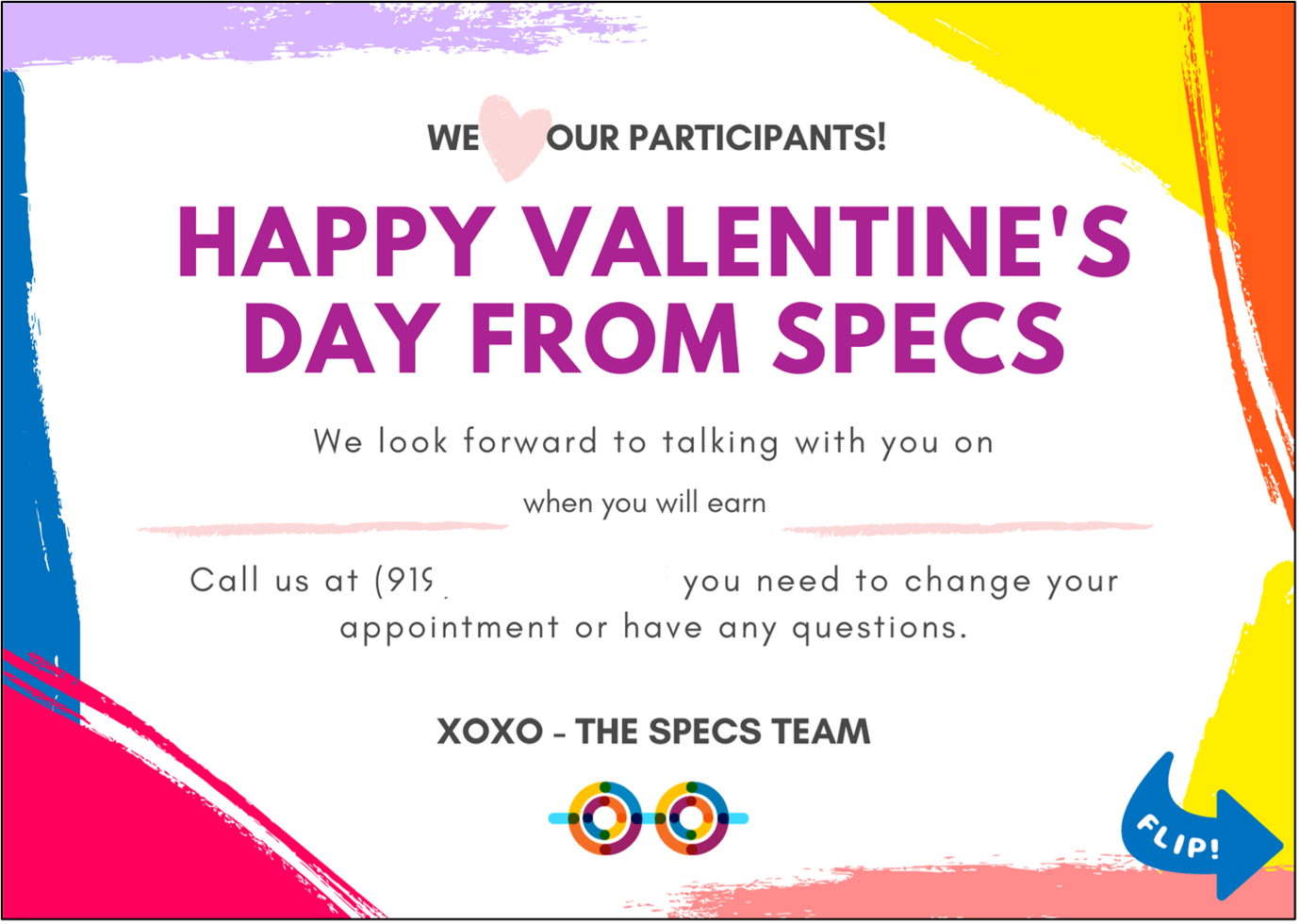

Supplement: S1 Appendix — (DOCX) [file pone.0283621.s001.docx]
