# Supplementary material for: Retention strategies among those on community supervision in the South: Lessons learned during the COVID-19 pandemic
Source: PLoS One. 2023 Apr 5;18(4):e0283621. doi: 10.1371/journal.pone.0283621 (PMC10075476; doi:10.1371/journal.pone.0283621)
Supplement: S2 Appendix — (DOCX) [file pone.0283621.s002.docx]

# **S4 Appendix: Retention Scripts**

**Script 1: Introducing retention bonuses**

We want to make sure that folks can stay engaged with our team. We are adding in an incentive of $5 if folks check in with us between study visits. You can call us up to two times before your visit on [DATE] for a total bonus of $10. At these check-ins you can update your contact information or just share with us how you are doing-- we just want to hear from you! Please call [SPECS number] to check in.

We also want to make sure folks can get their meetings completed in a timely manner. We are now offering $5 in addition to the scheduled $40 if you give us a call within the week before or after your scheduled meeting on [DATE]. You can also receive this incentive if you call us back within the first week after SPECS research assistants call you to complete your meeting.

We look forward to speaking to you on [DATE]. Call us at [SPECS number] if you have any questions.

**Script 2: Introducing contact payments**

We wanted to make you aware of a small change to the study that will help us locate individuals in our study in case they become hard to reach. If we become unable to reach a participant, we may incentivize their contacts with a limited-time payment to help us get back in touch with the participant. This is only to make sure that we don’t lose track of individuals whose contact information changes.

This change does not affect your compensation or enrollment in the study. If we need to reach out to your contacts, we will only be disclosing that we are calling from [school institution] and share with them that you are part of a health study or empowerment study. Do you have a preference?  We will NOT be sharing with your contacts:  that this is a paid study, how much money you can earn over the course of the study, we are not sharing anything about your criminal justice status (current or past), and we will NEVER share what you share with us when we complete the SPECS surveys, that information is private and confidential.

Do you have any questions about this policy change?

**Script 3: Engaging a contact:**

Hi, it is [name] from [school institution]. We are reaching out to you about [name of SPECS participant]. He/She/They are involved in our health study/resource program.  We really want to reach them and see how they are doing but are not having any success with the contact information we have. They listed you as a backup contact in case we couldn’t get in touch. Before COVID/coronavirus we would go out to their house and try and chat with them, but it is harder during COVID/coronavirus so we just got approved by [school institution] to pay $15.00 to [name of SPECS participant]’s contacts that [participant] provided IF they can put us in contact with [participant].

I know it is an inconvenience, but it would really help us if you can help me get in contact with [specs study participant name] and we can pay $15 for your effort.

Do you think you could help us get in contact with [participant]?

[If yes:] Great, thank you so much for your help. I just want to be clear about how this will work. When you have time, get in touch with them by phone, text, or in person and tell them that the SPECS Program has been trying to reach them and that we asked them to get in touch with us. Then, please pass along our phone number.

Do you have something to write this down with? [Provide SPECS Number]

Now, if [participant] gets in contact with us after you pass along our information, we will mail you $15 cash. Would you mind providing me a name and mailing address that is good to mail to?

Great. I want to be clear; we are only able to pay you if you successfully link us with [participant]. If [participant] doesn’t get in contact with us, we will have no way of knowing if you linked us with them. Know, that they do not have to continue with the program for you to be paid, but they should contact us to tell us they would like to stop.

Do you have any questions for us?

Finally, please do not harass or pester [participant] into calling us back if they don’t want to. If they are truly uninterested in continuing with the program and you have asked them more than twice, simply call us back and tell us what happened, and we will still be able to pay you.

Thank you for your help!
