# Supplementary material for: Retention strategies among those on community supervision in the South: Lessons learned during the COVID-19 pandemic
Source: PLoS One. 2023 Apr 5;18(4):e0283621. doi: 10.1371/journal.pone.0283621 (PMC10075476; doi:10.1371/journal.pone.0283621)
Supplement: S1 File — (PDF) [file pone.0283621.s003.pdf]

Locator Form Script

As I mentioned before, this study is for a total of 18 months, which is a long time and so throughout the study we will be asking you to fill out these "locator forms" so that we can be sure to have the most up-to-date ways of contacting you. We recognize that cell phone numbers change, phones run out of minutes, addresses change, even friends can change. So, we want to make sure we capture all the different ways you'd like to be contacted. This way we can remind you of study visits and you can continue to get paid for your participation in this study.

At the very end of this activity, I will ask for your Top 3 ways, you'd like to be contacted. I promise I will reach out to those 3 first and try for a few days before moving to other contacts. ALSO, if I speak to someone other than you know that I will never disclose you are 1) in a study; 2) share that it is a paid study; or 3) mention anything you've ever shared with me during study visits. I will only let them know I am from UNC and ask to give you the message that I called. If someone pushes to know more I may share that you are part of an "Empowerment Project at UNC" - would that work for you?
[If not okay for participant, ask for alternative or ask to keep to UNC-only]

Alternative Project Name

On here I ask about some specific things like, where you go to church. Know that I won't show up in the back of the church during service! Instead I may leave a sealed letter with a pastor or admin. Again, I will always do the Top 3 list you give me first or if you don't want me to go there then that's ok too, just don't give me that info.

If your Top 3 contacts aren't working, we will revisit those the next time we see each other so we can keep up with your preferences.

If I can't get in touch with you, I may mail letters to the addresses provided on this form. The letter would be addressed to you and would only say basically, "this is [name] from SPECS, give us a call."

Any questions before we being?

UNC-CH Locator Form

RA Name

Study Visit (Select One): ◯ Baseline ◯ 6 - Month ◯ 12 - Month ◯ 18 - Month Monthly Call # Date:

Full Legal Name: First MI Last Nickname/Alias

Preferred Name: DOB (mm/dd/yy):

DOC Number:

Home Phone: ☐ Voicemail?

Email Address(es):

Primary CellPhone: ☐ Voicemail?

Secondary Cell Phone: ☐ Voicemail?

Social Media: Facebook Twitter Instagram

Snapchat WhatsApp Other

Address paroled to: Street (PO Box, only if necessary) Apt. #

City State Zip Code

(IF NO PERMANENT ADDRESS, SKIP TO #4)

Permanent Address: Street (PO Box, only if necessary) Apt. #

City State Zip Code

1. How long have you lived at the above permanent address?

2. Do you plan to move any time soon? ☐ Yes ☐ No If yes, when?

New Address (If known): Street (PO Box, only if necessary) Apt. #

City State Zip Code

3. Does anyone else live with you? ☐ Yes ☐ No

First MI Last Relationship

First MI Last Relationship

First MI Last Relationship

First MI Last Relationship

ASK #4 ONLY IF NO PERMANENT ADDRESS

4. Is there a place where you live most of the time? ☐ Yes ☐ No

Location Name:

Address:

Street (PO Box, only if necessary) Apt. #

City State Zip Code

Days and times you may be there:

Who lives here?

5. Do you have a spouse or partner ? ☐ Yes ☐ No ☐ N/A

Name:

First MI Last Relationship

Address:

Street (PO Box, only if necessary) Apt. #

City State Zip Code

Phone(s): Email(s):

6. Are there any other family or friends who usually know how to reach you? ☐ Yes ☐ No

Contact #1

Name:

First MI Last Relationship

Address:

Street (PO Box, only if necessary) Apt. #

City State Zip Code

Phone(s): Email(s):

Contact #2

Name:

First MI Last Relationship

Address:

Street (PO Box, only if necessary) Apt. #

City State Zip Code

Phone(s): Email(s):

7. Are you still on parole? ☐ Yes ☐ No

StartDate(mm/yy): EndDate(mm/yy):

8. Is there a case worker, doctor, community clinic, pharmacy, or other contact that you see regularly who we could reach out to in order to contact you, if necessary? ☐ Yes ☐ No

Name: ______ First ______ MI ______ Last ______ Relationship

Address: ______ Street (PO Box, only if necessary) ______ Apt. #

______ City ______ State ______ Zip Code

Phone(s): ______ Email(s): ______

9. Do you currently have a job? ☐ Yes ☐ No ☐ N/A

Employer: ______

Address: ______ Street (PO Box, only if necessary) ______ Apt. #

______ City ______ State ______ Zip Code

Phone(s): ______ Email(s): ______

Days and times you may be there: ______

May we contact you at this location? ☐ Yes ☐ No ☐ N/A

10. Do you attend a religious institution regularly (e.g., church/ mosque/ synagogue)? ☐ Yes ☐ No ☐ N/A

Name of pastor or person of trust: ______

Address: ______ Street (PO Box, only if necessary) ______ Apt. #

______ City ______ State ______ Zip Code

Phone(s): ______ Email(s): ______

Days and times you may be there: ______

11. Is there a place you go regularly to hang out or meet friends? ☐ Yes ☐ No

Location: ______

Address: ______ Street (PO Box, only if necessary) ______ Apt. #

______ City ______ State ______ Zip Code

Phone(s): ______ Email(s): ______

Days and times you may be there: ______

12. Is there any other person we can contact or place we could visit in order to contact you, if necessary? ☐ Yes ☐ No

13. Thank you for providing that information. Of the contact methods that you have provided, which would be the <u>top three ways</u> to get in touch with you for the study?

1.

2.

3.

14. If we were to mail you payment for your participation in the study or any other study materials, which address would be the best to send this to?

Street (PO Box, only if necessary)	Apt. #

City	State	Zip Code

Does anyone else live with there? ☐ Yes ☐ No

First	MI	Last	Relationship
First	MI	Last	Relationship
First	MI	Last	Relationship
