# Supplementary material for: Retention strategies among those on community supervision in the South: Lessons learned during the COVID-19 pandemic
Source: PLoS One. 2023 Apr 5;18(4):e0283621. doi: 10.1371/journal.pone.0283621 (PMC10075476; doi:10.1371/journal.pone.0283621)
Supplement: S1 Table — (DOCX) [file pone.0283621.s004.docx]

| Study-branded Items Used in this Study | Item | Notes | Other study branded ideas | Item | Notes |
| --- | --- | --- | --- | --- | --- |
|  | Drawstring backpack | Great to provide a study-branded bag to hold participant's study appointment card; this is also useful for doing retention drop offs, a letter and additional swag can be placed in the bag and left outside doors if participants are not home. |  | mini toothbrush kit | Based on interactions study staff were having across sites with participants who were experiencing houselessness, there was an interest in investing in study promotion material that would be practically useful for that demographic; this included blankets and towels, menstrual product or hygiene kits, and toothbrush kits. |
|  | Hand sanitizer | Highly relevant during COVID-19. |  | N-95 Masks | Highly relevant during COVID-19. |
|  | Magnets |  |  | Roll up blanket w/ logo |  |
|  | Magnetic memo white board and dry erase marker | Great visual for a participant OR participant's contact to place on the refrigerator and be reminded of the study often. |  | Travel size hygiene kit (toothbrush, mouthwash, deodorant, lotion, soap, comb, menstrual products, etc.) |  |
|  | Resistance band |  |  | Bath towel w/ logo |  |
|  | Chapstick |  |  | Mini cool-down towel w/ logo |  |
|  | Snacks | Having water/snacks around during retention meetings can be really helpful |  | Long or short sleeve shirt w/ logo |  |
|  | Tissues |  |  | Socks/gloves with logo |  |
|  | Condoms |  |  | mini flashlight |  |
