# Supplementary figures and images for: Retention strategies among those on community supervision in the South: Lessons learned during the COVID-19 pandemic
Source: PLoS One. 2023 Apr 5;18(4):e0283621. doi: 10.1371/journal.pone.0283621 (PMC10075476; doi:10.1371/journal.pone.0283621)

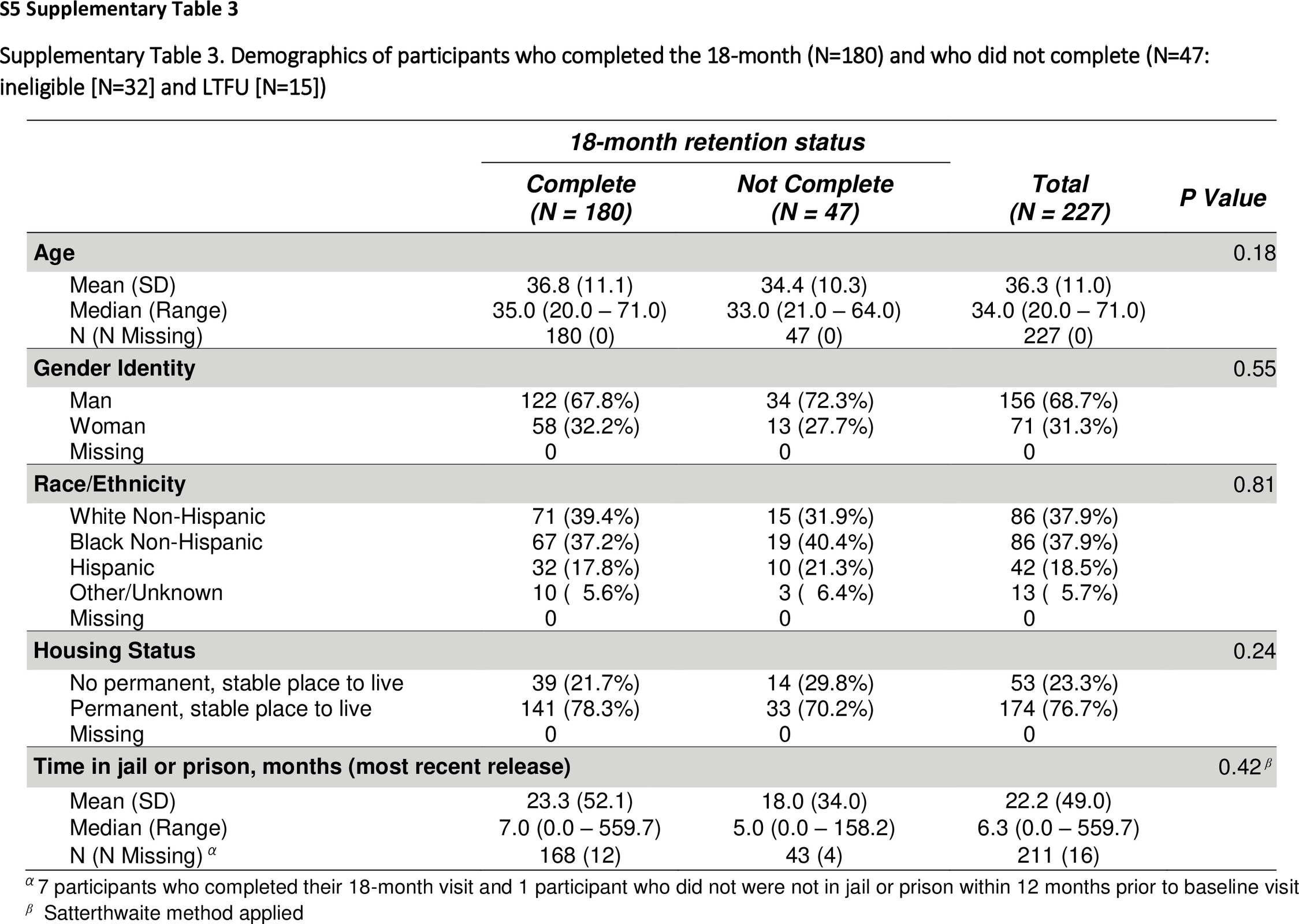

Supplement: S2 Table — (TIF) [file pone.0283621.s005.tif]
